# A Case of Hydrocele Stone with Its Composition Analysis

**DOI:** 10.1155/2010/586204

**Published:** 2010-06-14

**Authors:** Masayoshi Zaitsu, Koji Mikami, Yuta Takeshima, Takumi Takeuchi

**Affiliations:** Department of Urology, Kanto Rosai Hospital, 1-1 Kizukisumiyoshi-cho, Nakahara-ku, Kawasaki 211-8510, Japan

## Abstract

Hydrocele stones are freely mobile calcified bodies lying between the tunica vaginalis layers, and they are relatively rare. We present here another case of hydrocele stone incidentally discovered when castration was being undergone for the endocrine treatment of prostate cancer. A 71-year-old man was diagnosed as stage D2 prostate cancer with his prostate-specific antigen 387 ng/ml. A white smooth stone of 11 mm in diameter was incidentally found moving freely in the right hydrocele space during castration. The hydrocele stone was of yellow hard center with white materials around it. Crystallographical analysis of the hydrocele stone by a infrared spectrophotometer showed that the center was composed of 64% calcium carbonate and 36% calcium phosphate, while the outer portion was protein. Our case is the fourth where crystallographical analysis was reported for hydrocele stones.

## 1. Introduction

Hydrocele stones are freely mobile calcified bodies lying between the tunica vaginalis layers and they are relatively rare. The first report was published in 1975 by Chatterjee [1]. So far there have been a limited number of cases reported [2–10]. We present here another case of hydrocele stone incidentally discovered when castration was being undergone for the endocrine treatment of prostate cancer.

## 2. Case Presentation

**Figure 1:**
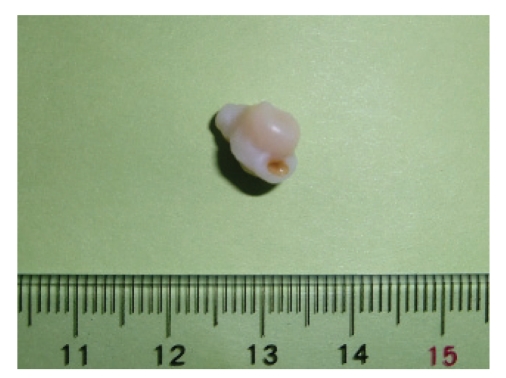
Photograph of hydrocele stone.

A 71-year-old man was presented with severe back pain to the orthopedic department, Kanto Rosai Hospital. Bone metastasis to Th1 of unknown origin was confirmed by CT scan, MRI, and bone scintigraphy. As his prostate specific antigen level was very high (387 ng/ml), he was referred to the urologic department. Digital rectal examination showed locally advanced prostate cancer. Transrectal ultrasound guided prostate biopsy and bilateral orchiectomy were performed on the same day in February, 2010. A smooth stone of 11 mm in diameter was incidentally found moving freely in the right hydrocele space (Figure 1). Bilateral testes (right 24 g, left 24 g) and epididymis were anatomically unremarkable, not indicating previous infection or trauma, but a little amount of serous liquid was drained from the space between the tunicae lining the scrotum and the testicles tunica vaginalis. Chemical, bacteriological, or cytological analysis of the hydrocele fluid was not performed. Intrascrotal abnormality had not been suspected before castration, thus imaging studies of intrascrotal space had not been done.

**Figure 2:**
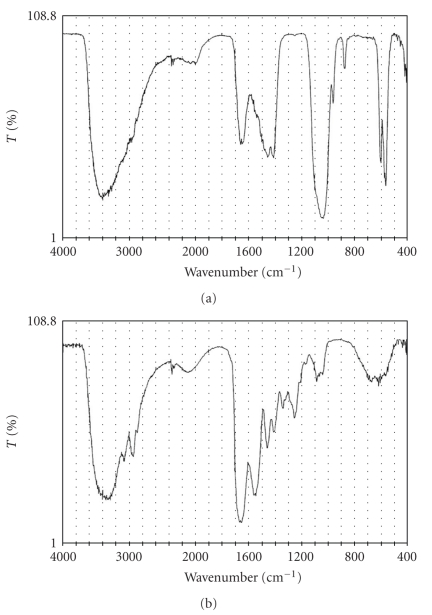
Infrared spectrophotometry of hydrocele stone (a) center of the stone, (b) outer of the stone.

Prostate biopsy revealed adenocarcinoma of Gleason score 7 in all four cores. Postoperative course was uneventful. The hydrocele stone was of yellow hard center with white matter around it. Crystallographical analysis of the hydrocele stone by an infrared spectrophotometer showed that the center was composed of 64% of calcium carbonate and 36% of calcium phosphate, while the outer portion was protein (Figure 2).

## 3. Discussion

Reported rates of calcification detected by ultrasound examination in hydrocele space were not very low (3 out of 863 patients [11] and 15 out of 350 patients [12]), we seldom encounter stones in the place during intrascrotal surgery, especially ones bigger in size. A calculus of 1.7 cm diameter attached to the parietal portion of the tunica vaginalis [9] and a calculus of 1.5 cm diameter attached to the head of the epididymidis [10] found in hydroceles were previously reported. Etiology for hydrocele stones is basically unknown. Preceding infection, hematoma, trauma, or torsion of appendix testis or appendix epididymis are suspected [11], but past history of that kind was not indicated in the present case.

The two reports indicating crystallography of calculi in hydrocele showed that three stones (2 mm, 2 mm, and 6 mm in size) in two cases were composed of hydroxyapatite (calcium phosphate ceramic: Ca_10_(PO_4_)_6_(OH)_2_) in the core surrounded by organic matters [4] and another stone which was very big (8.7 cm) in size was composed of magnesium ammonium phosphate [3]. Our case is the fourth where crystallographical analysis was reported for hydrocele stones and its composition is different from those of previously reported cases. Accumulation of such data must be significant in order to delineate the etiology of the disease.
